# Circular RNA EPHA3 suppresses progression and metastasis in prostate cancer through the miR-513a-3p/BMP2 axis

**DOI:** 10.1186/s12967-023-04132-4

**Published:** 2023-04-28

**Authors:** Huan Feng, Zhiyao Deng, Wei Peng, Xian Wei, Jihong Liu, Tao Wang

**Affiliations:** 1 Department of Urology, Tongji Hospital, Tongji Medical College, Huazhong University of Science and Technology, Wuhan, Hubei China; 2 Institute of Urology, Tongji Hospital, Tongji Medical College, Huazhong University of Science and Technology, Wuhan, Hubei China; 3 Shenzhen Huazhong University of Science and Technology Research Institute, Shenzhen, Guangdong China; 4 Department of Pediatric Surgery, Tongji Hospital, Tongji Medical College, Huazhong University of Science and Technology, Wuhan, Hubei China

**Keywords:** circEPHA3, miR-513a-3p, BMP2, Progression, Metastasis, Prostate cancer

## Abstract

**Background:**

Circular RNAs (circRNAs) may regulate the onset and progression of human malignancies by competitively binding to microRNA (miRNA) sponges, thus regulating the downstream genes. However, aberrant circRNA expression patterns and their biological functions in prostate cancer (PCa) warrant further studies. Our research sought to shed further light on the possible role and molecular mechanism of circEPHA3 action in controlling the growth and metastasis of PCa cells.

**Materials and methods:**

circEPHA3 (has_circ_0066596) was initially screened from a previous circRNA microarray and identified following Actinomycin D and RNase R assays. Fluorescence in situ hybridization, biotin-coupled probe RNA pulldown, and dual-luciferase reporter gene assays were performed to examine the relationship between circEPHA3 and miR-513a-3p. The biological role of circEPHA3 in PCa was assessed by CCK8, wound healing, Transwell assays, and animal experiments.

**Results:**

We identified a novel circular RNA, circEPHA3 (has_circ_0066596), which was down-regulated in high-grade PCa tissues and cell lines. The outcomes of CCK8, wound healing, Transwell assays, and animal experiments revealed that circEPHA3 prohibited the progression and metastasis of PCa in vivo and in vitro. Mechanistically, circEPHA3 was directly bound to miR-513a-3p and regulated the downstream gene, BMP2, thereby serving as a tumor suppressor in PCa.

**Conclusions:**

As a tumor suppressor, circEPHA3 inhibited the proliferation and metastasis of PCa cells through the miR-513a-3p/BMP2 axis, suggesting that circEPHA3 might be a potential therapeutic target for PCa.

**Supplementary Information:**

The online version contains supplementary material available at 10.1186/s12967-023-04132-4.

## Background

Prostate cancer (PCa) is the most prevalent malignancy among males. According to a recent study by the American Cancer Society, the incidence of PCa among males ranks first, accounting for 27% of the new cases, and its mortality rate ranks only second to that of lung cancer [1]. In recent years, the incidence and mortality of PCa have risen sharply in China, and the growth rate ranks second only to colorectal cancer [2]. The standard treatment approaches for PCa include surgery, radiation, endocrine therapy, and chemotherapy. Despite widespread screening for prostate-specific antigen, most PCa cases in China are typically detected at an advanced stage and the opportunity for surgery is lost. As the illness advances, a considerable proportion of patients show distant metastases, causing more than 400,000 deaths annually, anticipated to increase by more than double by 2040 [3]. Therefore, understanding the molecular processes that enhance PCa cell growth and metastasis is relevant.

Circular RNAs (circRNAs), novel non-coding RNAs generated by the reverse splicing of exons or/and introns of pre-mRNA transcripts [4], were first detected in the cytoplasm of eukaryotic cell lines in 1979 [5]. Due to the lack of a 5′ cap and 3′ polyadenylated tail [6], circRNAs are self-stable [7–9] and may progressively accumulate in certain cell types [10, 11], making them attractive diagnostic markers and biotherapeutic targets. Many studies have confirmed that circRNAs exhibit tissue-specific expression patterns [12–14], mainly operating as microRNA (miRNA) sponges, RNA-binding proteins, and protein-coding RNAs. For example, circSATB2 enhances the growth of non-small cell lung cancer cells by inhibiting the tumor suppressor activity of miR-326 [15]. circ-ZKSCAN1 modulates the miR-1178-3p/p21 axis, thus suppressing bladder cancer progression and lymph node metastasis [16]. circCDR1as inhibits glioma progression by disrupting the p53/MDM2 complex, thereby retaining p53 activity and protecting cells from DNA damage [17]. circZNF609 interacts with heavy multimers and is translated into proteins in a splicing-dependent manner [18]. circECE1 blocks the degeneration of c-Myc, further activating energy metabolism in osteosarcoma and boosting its incidence and progression [19]. Recently, some circRNAs have been found to show abnormal expression in PCa tissues. These circRNAs ultimately impact PCa development by regulating cellular proliferation, apoptosis, epithelial-mesenchymal transition, and chemoradiotherapy sensitivity [20–26]. The biological roles and regulatory pathways of PCa-related circRNAs are, however, largely unclear.

In the present study, we identified a novel circRNA, circEPHA3, using previous circRNA microarray data from human PCa tissues [22]. Next, we demonstrated that circEPHA3 was generated from exon 3 of the EPHA3 gene, and its expression was drastically suppressed in high-grade PCa tissues and androgen-independent cell lines. Furthermore, circEPHA3 could prevent the proliferation and metastasis of PCa cells in vivo and in vitro by interacting with miR-513a-3p to regulate BMP2 expression, opening up new avenues in the regulation of PCa development and metastasis.

## Material and methods

### Cell culture

Human PCa cell lines, 22Rv1, LNCaP, DU145, and PC-3 were procured from the China Center for Type Culture Collection (CCTCC, China) and grown in RPMI-1640 (Gibco, USA) media. HEK-293T cells were cultured in DMEM (Gibco, USA). Both RPMI-1640 and DMEM were supplemented with 10% fetal bovine serum (FBS, Gibco, USA) and 1% penicillin–streptomycin solution (Gibco, USA). All cell lines were maintained at 37 °C in a humidified incubator containing 5% CO_2_.

### DNA/RNA isolation and qRT-PCR

According to the specified protocols, genomic DNA (gDNA) and total RNA were extracted using FastPure Cell/Tissue DNA Isolation Mini Kit (Vazyme, China) and RNAiso Plus (Takara, Japan), respectively. PrimeScript RT Master Mix (Takara, Japan) was utilized for the reverse transcription of RNA to cDNA following the RNA quality test by NanoDrop 2000 (Thermofisher, USA). qRT-PCR was conducted on the QuantStudio 6 Flex (Applied Biosystems, USA) with Hieff® qPCR SYBR Green Master Mix (Yeasen, China). The sequences of relevant primers used in the study are listed in Additional file 1: Table S1.

### RNA stability assay

DU145 and PC-3 cells were treated with 5 μg/mL Actinomycin D (MedChemExpress, HY-17559, China) and harvested after 8, 16, and 24 h. For the RNase R assay, total RNA was treated with RNase R (Beyotime, R7092S, China) following the manufacturer’s instructions.

### Plasmid construction and transfection

circEPHA3 overexpression plasmids were constructed using human circEPHA3 cDNA and inserted in the plenti-ciR-GFP-T2A vector (IGE Biotechnology Co, China). After validation by sequencing, HEK-293T cells were transfected with the plasmids to package the lentivirus for infecting DU145 and PC-3 cells. After puromycin (2 μg/mL) selection for 7 days, surviving cells were considered stably transfected.siRNA was obtained from GenePharma (Shanghai, China). miRNA mimics, mimic control, miRNA inhibitor, and inhibitor control were purchased from RiboBio (Guangzhou, China). Lipofectamine RNAiMax (Life Technologies, USA) was utilized to transfect oligos into cells following the manufacturer’s instructions. The details of the sequences are shown in Additional file 2: Table S2.

### Cell proliferation, migration, and invasion assays

For the cell proliferation assay, 1500 cells/well were seeded in 96-well plates and treated with the Cell Counting Kit-8 (CCK-8; Boster, China) reagent. The absorbance of wells was measured at 450 nm on Multiskan FC (Thermo Scientific, USA). Three independent experiments were conducted.

Cell invasion and migration assays were performed using 8 µm pore size Transwell^®^ inserts (Corning, USA) coated with or without Matrigel (BD Science, USA). The plates were incubated at 37 °C for 24 h with 750 μL of RPMI-1640 medium supplemented with 10% FBS as a chemoattractant in the bottom chamber; 5 × 10^4^ cells were seeded in the upper chamber. After removing the inserts, noninvaded cells were wiped-off. Invaded cells to the bottom were fixed with methanol for 15 min and stained with 1% crystal violet solution. Three independent experiments were conducted.

### Wound healing assay

DU145 and PC-3 cells were seeded in 6-well plates, and wounds were generated at the center of the plates using 1 mL pipette tips. Floating cells were rinsed with PBS (Gibco, USA) and the remaining cells were grown in the serum-free RPMI-1640 medium. Cell migration was assessed by photographing the wound closure after 12 h. The wound closure area was measured using the ImageJ software. Three independent experiments were performed.

### Western blotting

All procedures were performed as described previously [27]. The primary antibodies against BMP2 (A0231) and β-actin (AC026) were purchased from ABclonal Biotechnology Co., Ltd. (China), and the secondary goat anti-rabbit antibody (BA1055) was procured from Boster Biotechnology Co., Ltd. (China). The pictures were captured and analyzed on the ChemiDoc Touch Imaging System (Bio-Rad, USA). Three independent experiments were conducted.

### Fluorescence in situ hybridization (FISH)

circEPHA3 and miRNA-513a-3p probes were constructed by GenePharma (Shanghai, China). The information about the probe is reported in Additional file 3: Table S3. These probes were identified using the RNA FISH Kit (GenePharma, China) based on specified protocols. All images were captured using the ZEISS LSM710 Confocal Microscope (Carl Zeiss, Germany).

### RNA pull-down assay

The circEPHA3 probes were treated for 2 h at room temperature with DynabeadsTM M-280 Streptavidin Magnetic Beads (Invitrogen, USA) to obtain probe-coated beads. The circEPHA3 overexpressing PCa cells were collected, lysed, sonicated, and treated overnight at 4 °C with probe-coated beads. After washing, RNAs were isolated using the RNAiso Plus (Takara, Japan) kit for qRT-PCR analysis. The sequences of probes are shown in Additional file 3: Table S3.

### Dual-luciferase reporter assay

HEK-293T cells were seeded into 12-well plates and transfected with luciferase plasmids in the pmirGlo vector (GenePharma, China) or miRNA mimics for 24 h. The Dual-Luciferase Reporter Assay System (Promega, USA) was used to assess the activities of the firefly and renilla luciferases. The ratio of the activity of the firefly to renilla luciferases denoted relative luciferase activity. Three separate tests were conducted.

### Haematoxylin and eosin (H&E) staining and immunohistochemistry (IHC)

The lung and tumor tissues were fixed for 48 h in 4% paraformaldehyde and embedded subsequently in paraffin. H&E and IHC staining assays were performed using the paraffin-embedded tumor tissue slices. The primary antibody against BMP2 (1:50, ABclonal, A0231, China) was used for staining. Images were acquired using the CaseViewer software (3DHISTECH Ltd., Hungary).

### Animal experiments

Four-week-old BALB/c nude mice were obtained from Jiangsu GemPharmatech Biotechnology Co., Ltd (China) and kept under specific pathogen-free conditions. The Ethics Committee on Animal Care of Tongji Hospital, Huazhong University of Science and Technology authorized all animal experiments. For xenograft experiments, circEPHA3 overexpressing or control PC-3/DU145 cells (5 × 10^5^ cells per animal) were transplanted subcutaneously into the left axilla of nude mice and tumor volumes were assessed weekly. The mice were euthanized four weeks later and the tumors were weighed. For the lung metastasis assay, 5 × 10^6^ PC-3/DU145 cells stably expressing luciferase were injected into the mice through their tail veins. Five weeks later, all mice were intraperitoneally injected with 150 mg/kg of D-luciferin and subsequently sedated with 3% Isoflurane (RWD Life Science, China) in an induction chamber for 15 min. The bioluminescence images were taken on an IVIS 100 Imaging System (Xenogen, Hopkinton, MA, USA), ventilated with 2.5% isoflurane. Each image was collected within 1 min. After the mice were sacrificed, the lung and tumor tissues were fixed and embedded in paraffin for H&E and IHC staining assays.

### Statistical analysis

GraphPad Prism 8.3.0 (GraphPad Prism, Inc., USA) was employed for all statistical analyses. To determine the statistical significance of differences between the two groups, the Student's t-test (two-tailed) was used. All data are shown as mean ± standard deviation. *P* < 0.05 was deemed statistically significant.

## Results

### Characterization of circEPHA3 expression in human PCa tissues and cell lines

**Fig. 1:**
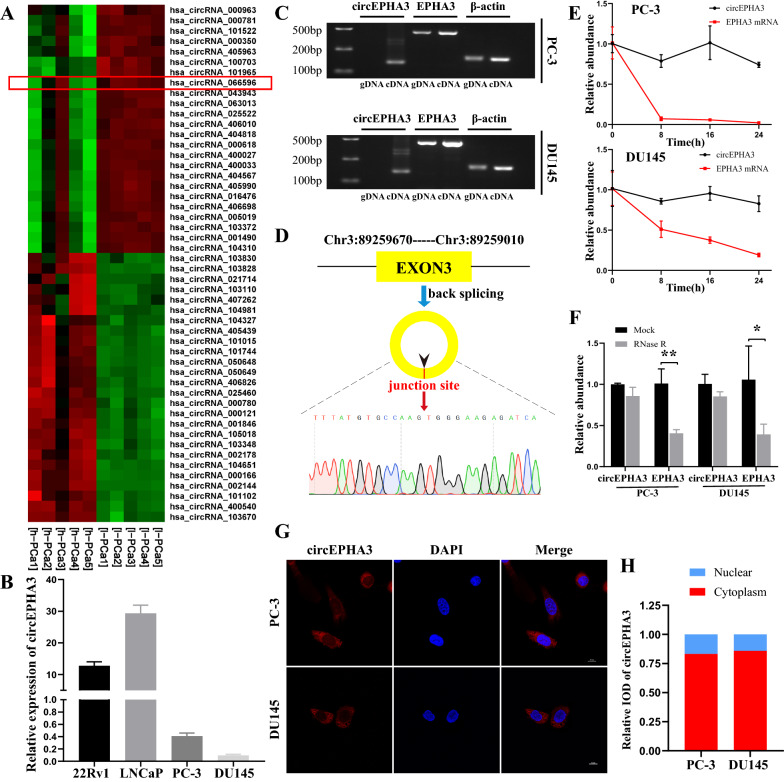
The identification and characterization of circEPHA3 in PCa. **A** Heat map of differentially expressed circRNA in high-grade and low-grade PCa tissues. (red: upregulation; green: downregulation; h-PCa: high-grade PCa; l-PCa: low-grade PCa). **B** Relative expression of circEPHA3 in various PCa cell lines. **C** The existence of circEPHA3 in PC-3 and DU145 cells. **D** The formation of circEPHA3 was confirmed by Sanger sequencing. **E** Relative abundance of circEPHA3 and EPHA3 mRNA in PC-3 and DU145 cells exposed to Actinomycin D. **F** Relative abundance of circEPHA3 and EPHA3 mRNA in PC-3 and DU145 cells in RNase R assay. **G** The subcellular localization of circEPHA3 in PCa cells was detected by FISH. Scale bars = 10 μm. **H** Relative integral optical density (IOD) of circEPHA3 in PCa cell. *, ** and *** represented *P* < 0.05, 0.01 and 0.001, respectively

Initially, we reviewed the circRNA microarray data from PCa tissues [22] and found its significant up-regulation in low-grade PCa compared to high-grade PCa cases (Fig. 1A). The level of circEPHA3 expression was dramatically down-regulated in the highly malignant PCa cell lines, PC-3 and DU145, compared to 22Rv1 and LNCaP cells (Fig. 1B). Since circRNAs are formed from their corresponding linear transcripts with the same sequence by back splicing, we designed convergent primers for EPHA3 mRNA and divergent primers for circEPHA3 according to their sequences. cDNA and gDNA were used as templates and qRT-PCR products were detected by agarose gel electrophoresis. circEPHA3 could only be identified in the cDNA from PC-3 and DU145 cells, whereas EPHA3 was amplified from both cDNA and gDNA (Fig. 1C). Next, Sanger sequencing analysis was performed for the products of qRT-PCR to verify the back splicing of circEPHA3. circEPHA3 was generated from exon 3 of the EPHA3 gene since the sequence of the amplified product matched that of circEPHA3 in circBase [28] (Fig. 1D).

Subsequently, the half-life of circEPHA3 was determined to be much longer than that of EPHA3 transcript following Actinomycin D exposure, indicating that circEPHA3 was more stable than EPHA3 (Fig. 1E). Simultaneously, the RNase R assay revealed that circEPHA3 could withstand RNase R digestion, whereas EPHA3 was mostly degraded (Fig. 1F). We identified the subcellular localization of circEPHA3 in PCa cells by FISH assay and found its distribution mainly in the cytoplasm (Fig. 1G–H). Generally, the findings indicated that circEPHA3 existed stably in PCa cells and was primarily localized in the cytoplasm of PC-3 and DU145 cells.

### circEPHA3 prevents the proliferation, migration, and invasion of PCa cells in vitro

**Fig. 2:**
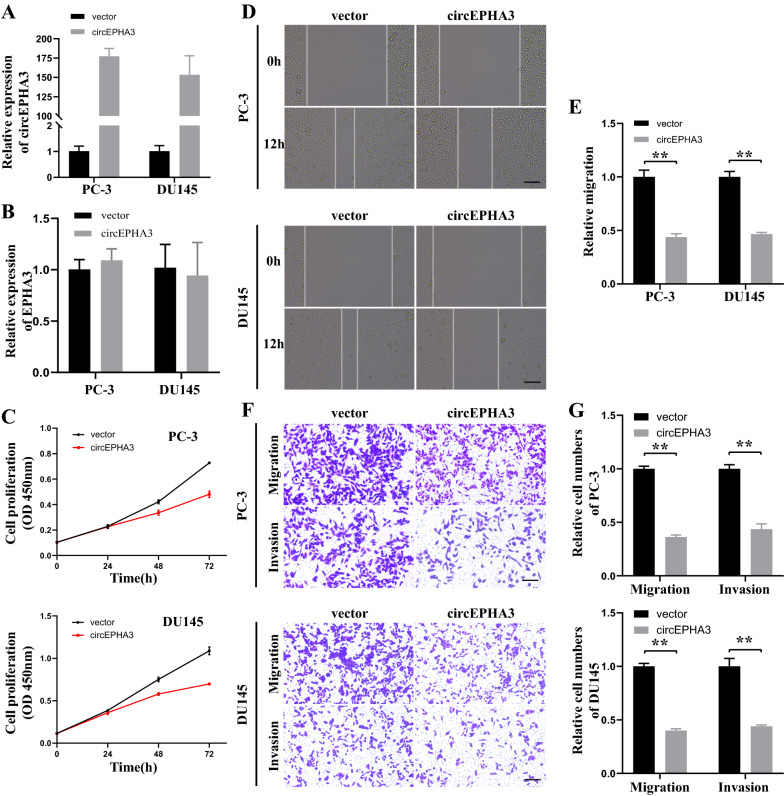
CircEPHA3 prevents the progression of PCa in vitro. **A, B** Relative expression of circEPHA3 and EPHA3 mRNA in PCa cells transfected with circEPHA3 or vector lentivirus. **C** The proliferative capacity of circEPHA3-overexpressed or vector PC-3 and DU145 cells was measured by CCK-8. **D, E** The migratory capacity of circEPHA3-overexpressed or vector PC-3 and DU145 cells was evaluated by wound healing assays. Scale bars = 100 μm. **F, G** The migration and invasion capabilities of circEPHA3-overexpressed or vector PC-3 and DU145 cells were assessed by transwell migration and invasion assays. Scale bars = 100 μm. *, ** and *** represented *P* < 0.05, 0.01 and 0.001, respectively

To examine the potential biological effects of circEPHA3 in PCa cells, we constructed circEPHA3 overexpressing PC-3 and DU145 cell lines and verified the transfection efficiency of circEPHA3 by qRT-PCR analysis (Fig. 2A). The expression of EPHA3 remained unaffected following the overexpression of circEPHA3 (Fig. 2B). Overexpressed circEPHA3 dramatically impaired the proliferative ability of PC-3 and DU145 cells (Fig. 2C). The wound healing assay revealed that overexpressed circEPHA3 hindered the migration of PC-3 and DU145 cells (Fig. 2D–E). Up-regulation of circEPHA3 significantly suppressed the migration and invasion of PCa cells, as evidenced by the Transwell assay (Fig. 2F–G). Therefore, our results demonstrated that circEPHA3 suppressed the proliferation, migration, and invasion of PCa cells.

### circEPHA3 bound to miR-513a-3p is a miRNA sponge in PCa cells

**Fig. 3:**
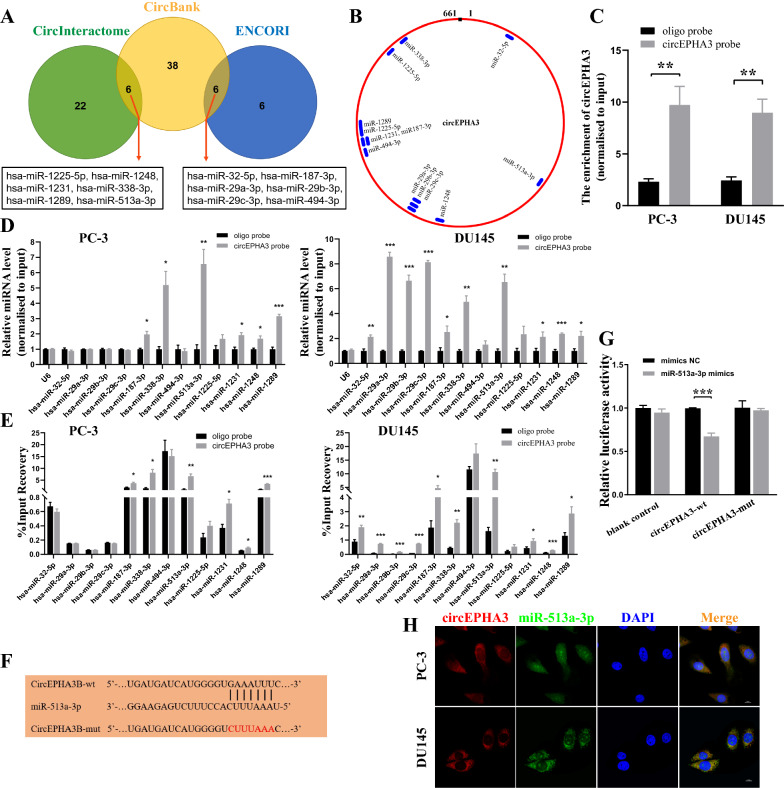
CircEPHA3 directly binds to miR-513a-3p in PCa cells. **A** CircBank, CircInteractome, and ENCORI have predicted twelve candidate circEPHA3 target miRNAs. **B** The schematic model displayed the preferred binding sites of circEPHA3 binding to 12 miRNA candidates. **C** Relative abundance of circEPHA3 in PC-3 and DU145 cells after RNA pull down. **D** Relative abundance of candidate miRNAs in PCa cells pulled down by biotinylated probes. **E** The percentage of input recovery of candidate miRNAs in PC-3 and DU145 cells pulled down by different probes. **F** The schematic of wild-type and mutant circEPHA3 luciferase reporter vectors. **G** Relative luciferase activity of HEK-293 T cells co-transfected with miR-513a-3p mimics or mimics NC, pmirGlo-wild type circEPHA3 (circEPHA3-wt) or pmirGlo-mutant type circEPHA3 (circEPHA3-mut) plasmids. **H** The FISH assay visualized the localization of circEPHA3 and miR-513a-3p in PCa cells. Scale bars = 10 μm. *, ** and *** represented *P* < 0.05, 0.01 and 0.001, respectively

Given that cytoplasmic circRNAs often function as miRNA sponges [29, 30], we attempted to check whether circEPHA3 could interact with miRNAs. We obtained 12 candidate miRNAs of circEPHA3 based on the predictions of CircBank [31], CircInteractome [32], and ENCORI [33], including miR-1225-5p, miR-1248, miR-1231, miR-338-3p, miR-1289, miR-513a-3p, miR -32-5p, miR-187-3p, miR-29a-3p, miR-29b-3p, miR-29c-3p, and miR-494-3p (Fig. 3A, B). Next, we designed a biotinylated circEPHA3 probe and oligo probe for the RNA pull-down assay. The efficacy of these probes was validated in PCa cells (Fig. 3C). Six miRNAs (miR-187-3p, miR-338-3p, miR-513a-3p, miR-1231, miR-1248, and miR-1289) were pulled down by the circEPHA3 probe in both PCa cell lines (Fig. 3D). The percentage of input recovery denoted the miRNA binding rate and the miRNA could potentially bind to circEPHA3 stably at %input recovery > 5 (Fig. 3E). Thus, only miR-513a-3p was abundantly pulled down by the circEPHA3 probe in PCa cells.

To further establish the binding of circEPHA3 and miR-513a-3p, we performed a dual-luciferase reporter assay. The pmirGlo vector, comprising the wild-type circEPHA3 sequence or the mutant miR-513a-3p binding region, was co-transfected with miRNA mimics in HEK-293T cells for 24 h (Fig. 3F). Up-regulation of miR-513a-3p significantly reduced the relative luciferase activity, implying that miR-513a-3p could efficiently bind to circEPHA3 (Fig. 3G). Furthermore, a FISH assay was conducted to investigate the interaction between circEPHA3 and miR-513a-3p, revealing their co-localized in the cytoplasm of both PC-3 and DU145 cells (Fig. 3H). Taken together, our data indicated that circEPHA3 might act as a competing endogenous RNA by sponging miR-513a-3p.

### miR-513a-3p promotes PCa cell progression by targeting BMP2

**Fig. 4:**
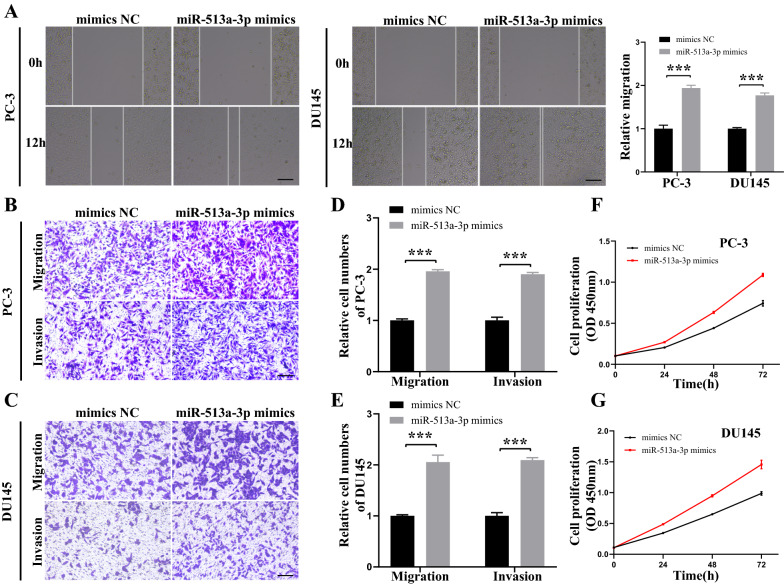
miR-513a-3p promotes PCa cells progression in vitro. **A** The migration capability of PC-3 and DU145 cells transfected with miR-513a-3p mimics or mimics NC was measured by wound healing assays. Scale bars = 100 μm. **B–E** The migration and invasion capabilities of PC-3 and DU145 cells transfected with miR-513a-3p mimics or mimics NC were assessed by transwell migration and invasion assays. Scale bars = 100 μm. **F, G** The proliferation ability of PC-3 and DU145 cells transfected with miR-513a-3p mimics or mimics NC was evaluated by CCK-8 assay. *, ** and *** represented *P* < 0.05, 0.01 and 0.001, respectively

First, we evaluated the biological effects of miR-513a-3p on PCa cells. Wound healing and Transwell assay suggested that miR-513a-3p could enhance the migration and invasion abilities of PCa cells (Fig. 4A–E). Up-regulation of miR-513a-3p markedly enhanced the proliferative ability of PCa cells, as evidenced by the CCK-8 assay (Fig. 4F–G). In contrast, miR-513a-3p down-regulation dramatically inhibited the proliferation, migration, and invasion capacities of PCa cells (Additional file 5: Fig. S1).

**Fig. 5:**
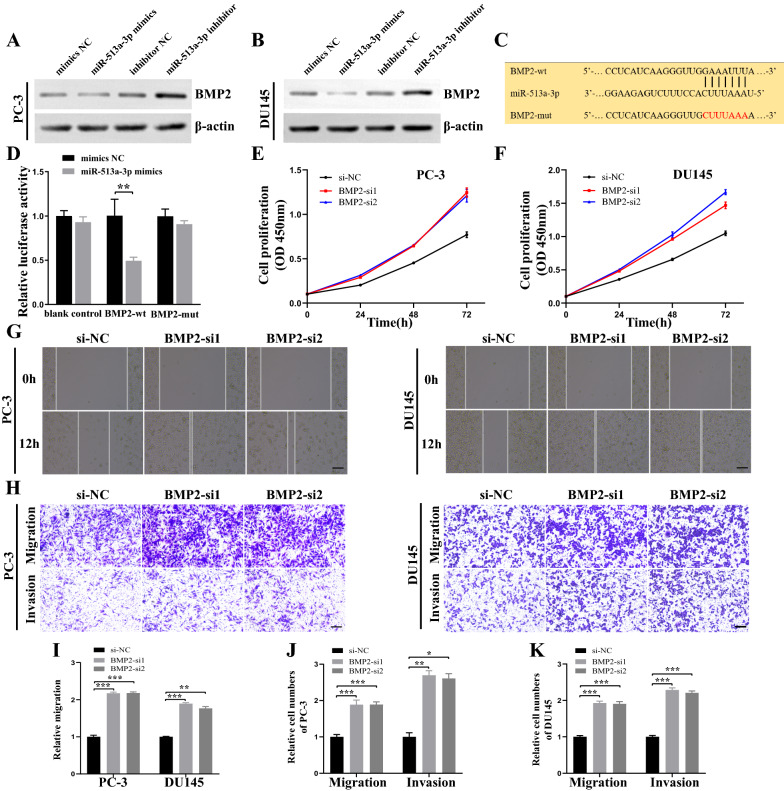
BMP2 is the downstream target of miR-513a-3p and promotes proliferation, migration and invasion of PCa cells in vitro. **A, B** The western blot showed that miR-513a-3p could down-regulate BMP2 protein expression in PC-3 and DU145 cells. **C** The schematic of BMP2 wild-type and mutant luciferase reporter vectors. **D** Relative luciferase activity of HEK-293T cells co-transfected with miR-513a-3p mimics or mimics NC, pmirGlo-wild type BMP2 (BMP2-wt) or pmirGlo-mutant type BMP2 (BMP2-mut) plasmids. **E, F** The proliferation ability of PC-3 and DU145 cells transfected with BMP2 siRNAs or negative control was evaluated by CCK-8 assay. **G, I** The migration capability of PC-3 and DU145 cells transfected with BMP2 siRNAs or negative control was measured by wound healing assays. Scale bars = 100 μm. **H, J–K** The migration and invasion capabilities of PC-3 and DU145 cells transfected with BMP2 siRNAs or negative control were assessed by transwell migration and invasion assays. Scale bars = 100 μm. *, ** and *** represented *P* < 0.05, 0.01 and 0.001, respectively

To investigate the downstream target of the circEPHA3/miR-513a-3p axis, RNA-seq analysis was conducted for circEPHA3 overexpressing and control PCa cells (Additional file 6: Fig. S2A, B). A total of 58 differentially expressed genes (DEGs) were identified in both PC-3 and DU145 cells (Additional file 6: Fig. S2C, D, Additional file 4). We next predicted the potential target genes of miR-513a-3p in PCa cells using TargetScan [34] and microT [35] (Additional file 6: Fig. S2E), and eventually obtained four candidate genes (MMD, COL3A1, BMP2, and SOX6). Subsequently, qRT-PCR analysis was performed. miR-513a-3p could not effectively regulate the expressions of SOX6, MMD, and COL3A1 (Additional file 6: Fig. S2F–H). Western blot analysis showed that miR-513a-3p mimics reduced the abundance of BMP2 protein, while miR-513a-3p inhibitor promoted its levels in both PCa cells, indicating that BMP2 might be the target gene of miR-513a-3p likely regulated by circEPHA3 (Fig. 5A, B, Additional file 7: Fig. S3A, B). To validate the above findings, we conducted a dual luciferase reporter assay with pmirGlo vectors harboring either wild-type or mutant sequences of BMP2. The relative luciferase activity decreased significantly in HEK-293T cells transfected with BMP2-wt 3′ untranslated region (3′ UTR) vector compared to HEK-293T cells transfected with the BMP2-mut vector (Fig. 5C, D).

Next, we designed siRNA constructs against BMP2 to evaluate its effect on PCa cells. Down-regulation of BMP2 stimulated the growth of PCa cells (Fig. 5E, F), and the migratory capacity of PCa cells was enhanced following treatment with BMP2 siRNAs (Fig. 5G, I). Moreover, Transwell assays demonstrated that silencing of BMP2 elevated cell migration and invasion of PCa cells (Fig. 5H, J–K). The aforementioned results suggested that BMP2 exerted an antitumor impact on PCa cells. Collectively, our findings revealed that miR-513a-3p performed an oncogenic function through BMP2.

### circEPHA3 inhibits the progression of PCa cells via miR-513a-3p/BMP2

**Fig. 6:**
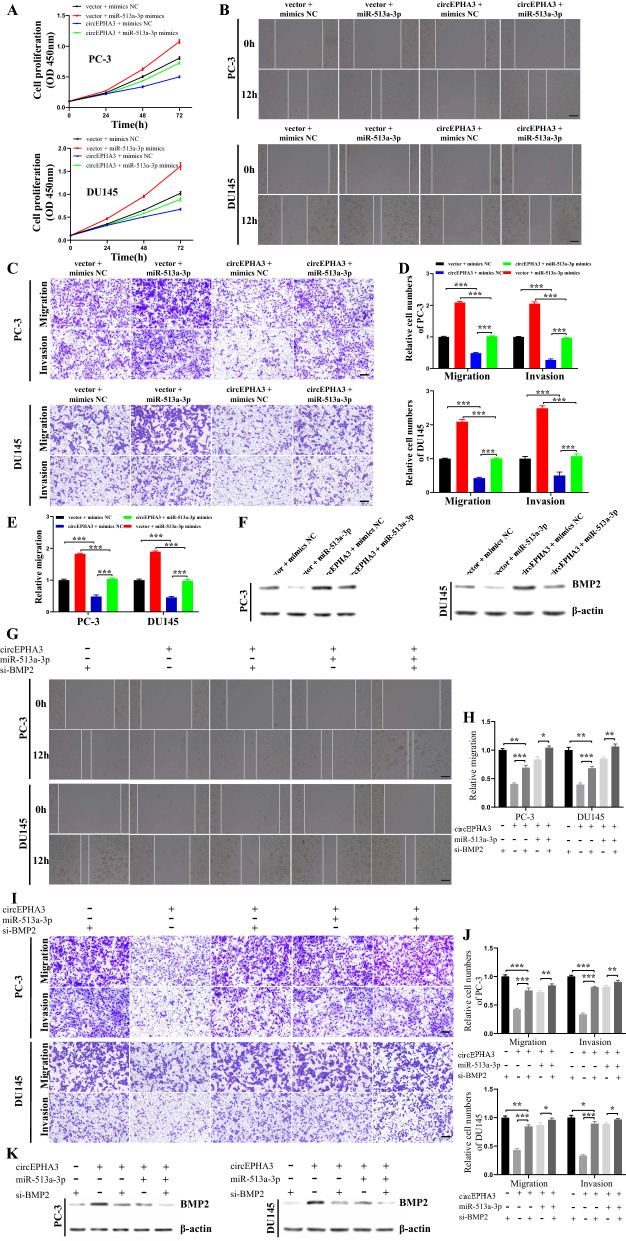
CircEPHA3 inhibits the migration and invasion of PCa cells via miR-513a-3p/BMP2. **A** Up-regulation of circEPHA3 abolished the cell proliferative capacity of PC-3 and DU145 cells enhanced by miR-513a-3p mimics. **B, E** The migration capability of PC-3 and DU145 cells transfected with miR-513a-3p mimics was counteracted by circEPHA3 in wound healing assays. Scale bars = 100 μm. **C, D** The migration and invasion capabilities of PC-3 and DU145 cells transfected with miR-513a-3p mimics was neutralized by circEPHA3 in transwell assays. Scale bars = 100 μm. **F** The western blot showed that circEPHA3 could reverse the effect of miR-513a-3p on BMP2 protein expression in PC-3 and DU145 cells. **G, H** The migration capability of circEPHA3-overexpressed PC-3 and DU145 cells co-transfected with miR-513a-3p mimics and/or BMP2 siRNA in wound healing assays. Scale bars = 100 μm. **I, J** The migration and invasion capabilities of PC-3 and DU145 cells co-transfected with miR-513a-3p mimics and/or BMP2 siRNA in transwell assays. Scale bars = 100 μm. **K** The western blot showed the level of BMP2 protein in PC-3 and DU145 cells that co-transfected with circEPHA3, miR-513a-3p and/or BMP2 siRNA. *, ** and *** represented *P* < 0.05, 0.01 and 0.001, respectively

Rescue experiments were performed to assess whether circEPHA3 functioned as a tumor suppressor in PCa cells by sponging miR-513a-3p. The proliferation ability of circEPHA3 overexpressing PCa cells transfected with miR-513a-3p mimics was impaired compared to those co-transfected with the vector and miR-513a-3p mimics, suggesting that up-regulation of circEPHA3 could partially abolish the miR-513a-3p-driven improvement in cellular proliferation (Fig. 6A). Overexpression of circEPHA3 could debilitate the promotion of migration and invasion of PCa cells mediated by miR-513a-3p (Fig. 6B–E). The level of BMP2 protein was higher in circEPHA3 overexpressing PCa cells transfected with miR-513a-3p mimics than vector PCa cells transfected with miR-513a-3p mimics (Fig. 6F; Additional file 7: Fig. S3C, D). Taken together, circEPHA3 could abolish the carcinogenic impact of miR-513a-3p on PCa cells.

Subsequently, knockdown of BMP2 in circEPHA3-overexpressed PCa cells significantly enhanced the migration and invasion, and circEPHA3 could inhibit the migration and invasion of PCa cells induced by BMP2 knockdown. Meanwhile, it has been pointed out above that upregulation of miR-513a-3p reversed the inhibitory effect of circEPHA3 on PCa cells, based on which the migration and invasion ability of PCa cells were boosted by knocking down BMP2 (Fig. 6G–J). In addition, it was revealed that the expression level of BMP2 protein could be co-regulated by circEPHA3/miR-513a-3p axis (Fig. 6K; Additional file 7: Fig. S3E, F). Taken together, these results indicated that circEPHA3 could suppress the progression of PCa cells through the miR-513a-3p/BMP2 pathway.

### cirEPHA3 suppresses tumor growth and metastasis in vivo

**Fig. 7:**
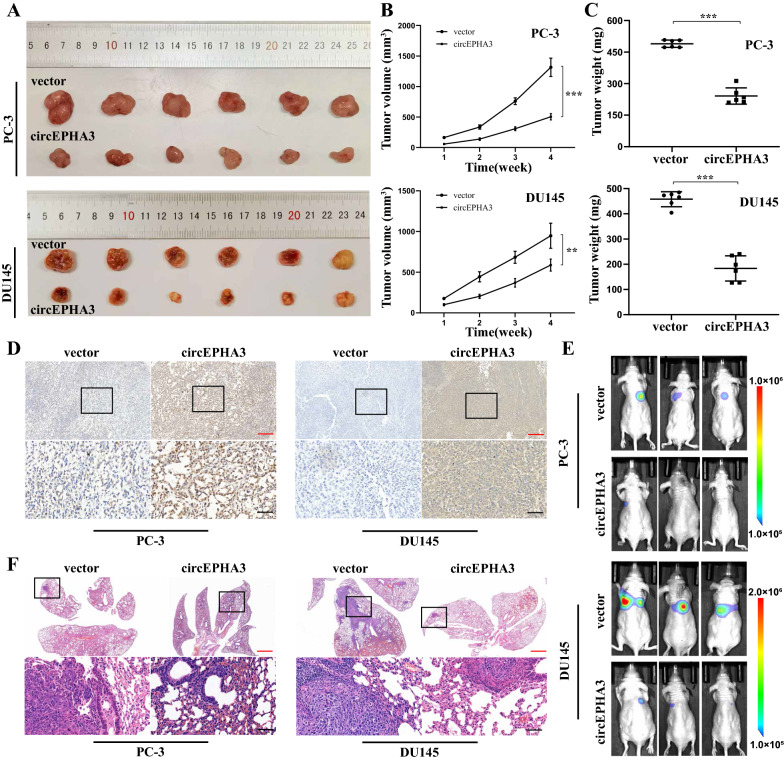
CircEPHA3 prevents tumor growth and metastasis in vivo. **A** The subcutaneously xenograft tumors formed by circEPHA3-overexpressed or vector PCa cells in BALB/c nude mice. **B, C** The tumor volume and weight of circEPHA3-overexpressed and vector PCa groups. **D** The expression of BMP2 protein in circEPHA3-overexpressed and vector PCa groups was assessed by IHC analysis. The red scale bars = 100 μm, the black scar bars = 20 μm. **E** Representative images of bioluminescence in both metastasis groups captured by IVIS 100 Imaging System. **F** Representative images of H&E of the lungs in circEPHA3-overexpressed and vector PCa groups. The red scale bars = 500 μm, the black scar bars = 20 μm. *, ** and *** represented *P* < 0.05, 0.01 and 0.001, respectively

To assess the impact of circEPHA3 expression on tumor growth in vivo, circEPHA3 overexpressing or negative control PC-3 and DU145 cells were subcutaneously implanted into BALB/c nude mice. In the circEPHA3-overexpressed group, tumor sizes and weights reduced considerably compared to the control group (Fig. 7A–C). The xenografted tumors in both groups were utilized for IHC analysis, and the level of BMP2 expression was significantly high in tumor tissues of the circEPHA3 overexpressing group (Fig. 7D). To examine the influence of circEPHA3 expression on tumor cell metastasis in vivo, we injected circEPHA3 overexpressing or the control PCa cells into the tail vein of mice to establish lung metastasis models. The findings revealed that the circEPHA3-overexpressed group's range of metastasis reduced notably and lung bioluminescence was weak or undetectable, suggesting that circEPHA3 might prevent PCa metastasis in vivo (Fig. 7E, F; Additional file 8: Fig. S4).

## Discussion

Recently, numerous circRNAs have been identified in eukaryotes owing to the advancement in high-throughput sequencing technology and bioinformatics [36–38]. Since circRNAs have been demonstrated as potential promising biomarkers, their distinct expression properties and crucial biological functions in various diseases, including cancer [39–41], cardiovascular diseases [42], neurological diseases [43], and autoimmune disorders [44] have been elucidated. In this investigation, we identified a novel circRNA in PCa, circEPHA3, which was significantly downregulated in high-grade PCa and highly aggressive PC-3 and DU145 PCa cell lines. circEPHA3 was first reported by Huang et.al, wherein it was drastically down-regulated in docetaxel-resistant breast cancer cell lines and probably affected docetaxel sensitivity by sponging miRNAs, as evidenced by bioinformatics analysis [45]. However, their findings have not been validated further. To date, this is the first study demonstrating the characteristics and regulatory roles of circEPHA3 in PCa.

In functional experiments, overexpression of circEPHA3 dramatically weakened the proliferation, migration, and invasion abilities of PCa cells. Moreover, to examine the impact of circEPHA3 on PCa in vivo, we employed circEPHA3 overexpressing and control PCa cell lines to develop a xenograft tumor model and lung metastasis model in nude mice. Interestingly, the growth and metastasis of tumors originating from circEPHA3 overexpressing PC-3 and DU145 cells were suppressed. These findings demonstrated that circEPHA3 was a tumor suppressor of PCa.

Numerous miRNA binding sites facilitate the interaction between circRNAs and miRNAs [7, 30]. For instance, circEPSTI1 competitively binds to miR-4753 and miR-6809, thus altering the proliferation of triple-negative breast cancer cells and apoptosis [46]. circACVR2A interferes with the cancer-promoting activity of miR-626 to inhibit the growth and lymph node metastasis of bladder cancer cells [47]. Evidence indicates that the cytoplasmic distribution of circRNAs is closely related to miRNA sponging [29, 30]. Herein, in the FISH assay, we observed that circEPHA3 was mostly localized in the cytoplasm of PCa cells. CircBank, CircInteractome, and ENCORI databases were employed to screen potential miRNAs. Finally, we confirmed that miR-513a-3p was the target of circEPHA3 by RNA pull-down and dual luciferase reporter gene assays. miR-513a-3p exhibited an oncogenic impact on PCa cells, as evidenced by the transfection of miR-513a-3p mimics and inhibitors into PC-3 and DU145. Moreover, rescue experiments revealed that circEPHA3 could reverse the promotion of progression and metastasis of PCa cells induced by miR-513a-3p. These findings showed that miR-513a-3p might be captured by circEPHA3 by its miRNA sponging.

Mature miRNAs primarily bind to the 3’ UTR of mRNAs, leading to the regulation of the target gene expression [48, 49]. Recent studies have demonstrated that circRNAs directly target miRNAs to prohibit them from regulating downstream target genes [15, 16, 22, 46, 47]. In this study, we performed RNA-seq analysis and utilized prediction tools (TargetScan and microT). BMP2 was determined as the candidate gene of the circEPHA3/miR-513a-3p axis, further validated by the results of the dual-luciferase reporter assay. BMP2 inhibits the proliferation and metastasis of tumor cells in breast cancer [50], colon cancer [51], and gastric cancer [52]. Two other comparative studies have indicated that low expression and absence of BMP2 might be related to progression to a more aggressive phenotype in PCa [53] and signal poor prognosis of patients with ovarian cancer [54]. In addition, a recent retrospective study demostrated that the low BMP2 expression was a significant predictor of biochemical recurrence (BCR) in PCa and the patients with low BMP2 expression had poorer BCR-free survival [55]. However, the biological role of BMP2 in PCa has not yet been confirmed. In this study, inhibition of BMP2 enhanced the proliferation, migration, and invasion of PCa cells, complementing previous findings. Subsequently, the cell function assays showed that circEPHA3 significantly impaired the migration and invasion of PCa cells promoted by BMP2 knockdown, while downregulation of BMP2 effectively enhanced the progression of PCa cells in the presence of co-transfection of circEPHA3 and miR-513a-3p. These results consistently implicated BMP2 as the downstream target of the circEPHA3/miR-513a-3p axis in the regulation of PCa cells biological behavior.

However, there are some weaknesses in this study. The study lacks sufficient clinical samples to further verify the regulatory role of cricEPHA3 in the progression and metastasis of prostate cancer at the clinical level. We will further improve this work in the next study, through the collection of appropriate clinical samples of prostate cancer to verify the feasibility of cricEPHA3 as a prognostic marker or treatment target.

## Conclusions

**Fig. 8:**
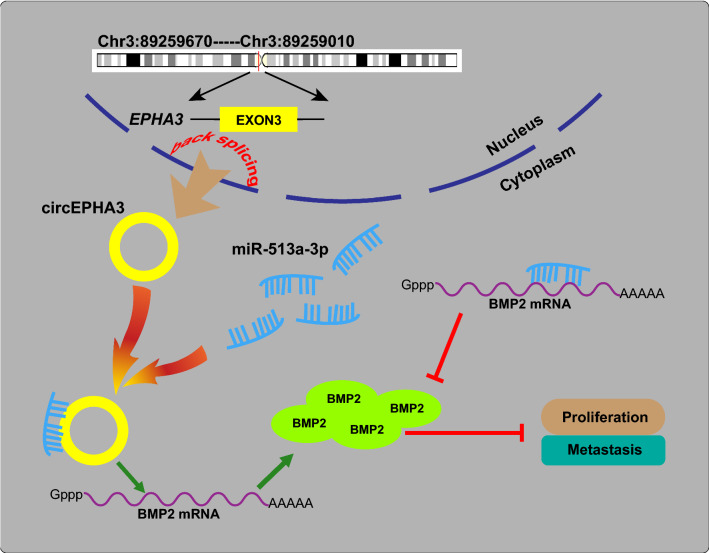
The schematic illustration of circEPHA3 inhibiting PCa cell proliferation and metastasis via the miR-513a-3p/BMP2 axis

In summary, our findings indicated that circEPHA3 expression was suppressed in high-grade tissues and highly malignant PCa cell lines. Mechanistically, circEPHA3 effectively suppressed the proliferation and metastasis of PCa cells by interacting with miR-513a-3p and counteracting the inhibitory impact of miR-513a-3p on BMP2 expression (Fig. 8). The circEPHA3/miR-513a-3p/BMP2 is a novel regulatory axis in PCa, which is expected to provide new insight into the treatment strategy against PCa progression and metastasis.

## Supplementary Information

**Additional file 1: Table S1.** The sequences of relevant primers used in qRT-PCR.

**Additional file 2: Table S2.** The oligonucleotides transfected in this study.

**Additional file 3: Table S3.** The probes used in FISH and RNA pull down.

**Additional file 4: **The 58 differentially expressed genes (DEGs) were screened and regulated in both PC-3 and DU145 cells after transfected circEPHA3 lentivirus.

**Additional file 5: Figure S1.** miRNA-513a-3p exerts carcinogenic impact on PCa cells in vitro. (A) Relative expression of miR-513a-3p in PCa cell lines. (B) The relative abundance of miR-513a-3p in PC-3 and DU145 cells transfected with miR-513a-3p mimics or inhibitor.** (C)** The migration capability of PC-3 and DU145 cells transfected with miR-513a-3p inhibitor or inhibitor NC was measured by wound healing assays. The magnification is 100×. **(D-G)** The migration and invasion capabilities of PC-3 and DU145 cells transfected with miR-513a-3p inhibitor or inhibitor NC were assessed by Transwell migration and invasion assays. The magnification is 100×. **(H, J)** The proliferation ability of PC-3 and DU145 cells transfected with miR-513a-3p inhibitor or inhibitor NC was evaluated by CCK-8 assay. All data are expressed as means ± standard deviation. *P < 0.05, **P < 0.01 and ***P < 0.001.

**Additional file 6: Figure S2.** The screening of downstream target genes of miRNA-513a-3p.** (A, B)** Heat map of DEGs between circEPHA3-overexpressed and vector PCa cells. **(C, D)** Circos of 58 regulated DEGs in both PC-3 and DU145 cells. **(E) **Four candidate downstream target genes of circEPHA3/miR-513a-3p were predicted by TargetScan, microT and RNA-seq DEGs.** (F-H)** Relative expression of SOX6, MMD and COL3A1 in PCa cells after transfection of miR-513a-3p mimics, inhibitor and corresponding negative control. All data are expressed as means ± standard deviation. *P < 0.05, **P < 0.01 and ***P < 0.001.

**Additional file 7: Figure S3.** **(A)** The relative expression of BMP2 in PC-3 cells transfected with miR-513a-3p mimics and inhibitor. **(B)** The relative expression of BMP2 in DU145 cells transfected with miR-513a-3p mimics and inhibitor. **(C) **The relative expression of BMP2 in PC-3 cells transfected with miR-513a-3p mimics and cricEPHA3.** (D)** The relative expression of BMP2 in DU145 cells transfected with miR-513a-3p mimics and cricEPHA3. **(E)** The relative expression of BMP2 in PC-3 cells co-transfected with circEPHA3, miR-513a-3p and/or BMP2 siRNA. **(F)** The relative expression of BMP2 in DU145 cells co-transfected with circEPHA3, miR-513a-3p and/or BMP2 siRNA. All data are expressed as means ± standard deviation. *P < 0.05, **P < 0.01 and ***P < 0.001.

**Additional file 8: Figure S4.** (**A**) The photon flux of each group of nude mice. (**B**) The relative invasion area of the metastatic tumor. All data are expressed as means ± standard deviation. **P < 0.01 and ***P < 0.001.

## Data Availability

The data that support the findings of this study are available from the corresponding author upon reasonable request.
