# Supplementary material for: Circular RNA EPHA3 suppresses progression and metastasis in prostate cancer through the miR-513a-3p/BMP2 axis
Source: J Transl Med. 2023 Apr 28;21:288. doi: 10.1186/s12967-023-04132-4 (PMC10148471; doi:10.1186/s12967-023-04132-4)
Supplement: Supplementary file 1 — Additional file 1: Table S1. The sequences of relevant primers used in qRT-PCR. [file 12967_2023_4132_MOESM1_ESM.docx]

| **Primers** | **Sequence (5’-3’)** |
| --- | --- |
| CircEPHA3 Forward | TGCAGTACAGAAGGCGAATG |
| CircEPHA3 Reverse | GTCCATGACATTGCACACCT |
| EPHA3 Forward | GGCTCTGACACCCTTATGTTG |
| EPHA3 Reverse | CCCTTCTTGTTGACAGGACC |
| β-actin Forward | CCTTCCTGGGCATGGAGTC |
| β-actin Reverse | TGATCTTCATTGTGCTGGGTG |
| miR-1225-5p Forward | GCCGAGGTGGGTACGGCCCA |
| miR-1248 Forward | ACCTTCTTGTATAAGCACTGTGCTAAA |
| miR-1231 Forward | TATGTGTCTGGGCGGACAG |
| miR-1289 Forward | CGTGGAGTCCAGGAATCTGC |
| miR-338-3p Forward | GGTCCAGCATCAGTGATTTTGTTG |
| miR-513a-3p Forward | GGGTAAATTTCACCTTTCTGAGAAGG |
| miR-32-5p Forward | GGCTATTGCACATTACTAAGTTGCA |
| miR-187-3p Forward | CGTGTCTTGTGTTGCAGCC |
| miR-29a-3p Forward | CCTAGCACCATCTGAAATCGGTTA |
| miR-29b-3p Forward | GGTAGCACCATTTGAAATCAGTGTT |
| miR-29c-3p Forward | GGTAGCACCATTTGAAATCGGTTA |
| miR-494-3p Forward | CTGAAACATACACGGGAAACCTC |
| Poly A RT | CTCTACAGCTATATTGCCAGCCACACTAATTTTTTTTTTTTTTT |
| Poly A Reverse | CTCTACAGCTATATTGCCAGCC |
| U6 Forward | CGCTTCGGCAGCACATATAC |
| U6 Reverse | TTCACGAATTTGCGTGTCAT |
| SOX6 Forward | ACGCCTGGAAGCATTTC |
| SOX6 Reverse | TCAGGGGCATACCTGTTTA |
| MMD Forward | ATGCGTTGGTTTATCTGGCTC |
| MMD Reverse | AGTCCATCGGTGTTGTTCATTG |
| COL3A1 Forward | GCCAAATATGTGTCTGTGACTCA |
| COL3A1 Reverse | GGGCGAGTAGGAGCAGTTG |

**Table S1.** **The sequences of relevant primers used in qRT-PCR.**
