# Supplementary material for: Circular RNA EPHA3 suppresses progression and metastasis in prostate cancer through the miR-513a-3p/BMP2 axis
Source: J Transl Med. 2023 Apr 28;21:288. doi: 10.1186/s12967-023-04132-4 (PMC10148471; doi:10.1186/s12967-023-04132-4)
Supplement: Supplementary file 2 — Additional file 2: Table S2. The oligonucleotides transfected in this study. [file 12967_2023_4132_MOESM2_ESM.docx]

| **Oligonucleotides** | **Sequence (5’-3’)** |
| --- | --- |
| si-NC sense | UUCUCCGAACGUGUCACGUTT |
| si-NC antisense | ACGUGACACGUUCGGAGAATT |
| BMP2-si1 sense | GCAGUUUCCAUCACCGAAUTT |
| BMP2-si1 antisense | AUUCGGUGAUGGAAACUGCTT |
| BMP2-si2 sense | GCUGUACCUUGACGAGAAUTT |
| BMP2-si2 antisense | AUUCUCGUCAAGGUACAGCTT |
| mimics NC sense | UUUGUACUACACAAAAGUACUG |
| mimics NC antisense | CAGUACUUUUGUGUAGUACAAA |
| miR-513a-3p mimics sense | UAAAUUUCACCUUUCUGAGAAGG |
| miR-513a-3p mimics antisense | CCUUCUCAGAAAGGUGAAAUUUA |
| inhibitor NC | CAGUACUUUUGUGUAGUACAAA |
| miR-513a-3p inhibitor | CCUUCUCAGAAAGGUGAAAUUUA |

**Table S2.** **The oligonucleotides transfected in this study.**
