# Supplementary material for: Circular RNA EPHA3 suppresses progression and metastasis in prostate cancer through the miR-513a-3p/BMP2 axis
Source: J Transl Med. 2023 Apr 28;21:288. doi: 10.1186/s12967-023-04132-4 (PMC10148471; doi:10.1186/s12967-023-04132-4)
Supplement: Supplementary file 3 — Additional file 3: Table S3. The probes used in FISH and RNA pull down. [file 12967_2023_4132_MOESM3_ESM.docx]

**Table S3. The probes used in FISH and RNA pull down.**

|  | **Sequence (5’-3’)** |
| --- | --- |
| **FISH Probes** | |
| Cy3-circEPHA3 | ACACCACTGATCTCTTCCCACTTGGCACAT AAAACCTCTT |
| Cy5-miR-513a-3p | CCTTCTCAGAAAGGTGAAATTTA |
| **RNA pull down probes** | |
| Biotin-NC | CACCCTGCCATAAAGAAACT |
| Biotin-circEPHA3 | ACACCACTGATCTCTTCCCACTTGGCACATAAAACCTCTTTCTTCATAGCCAGCA |
