# Supplementary figures and images for: Circular RNA EPHA3 suppresses progression and metastasis in prostate cancer through the miR-513a-3p/BMP2 axis
Source: J Transl Med. 2023 Apr 28;21:288. doi: 10.1186/s12967-023-04132-4 (PMC10148471; doi:10.1186/s12967-023-04132-4)

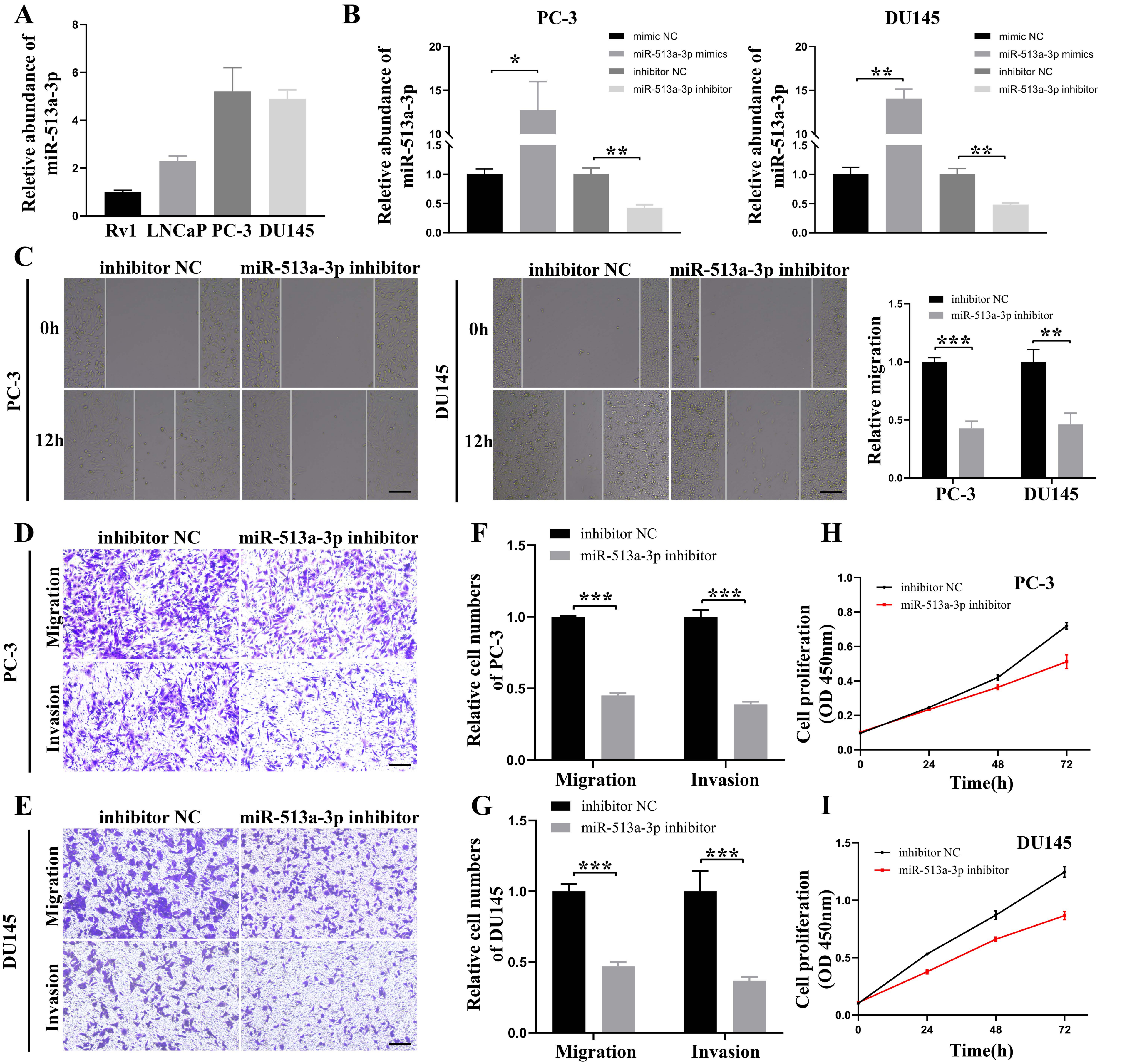

Supplement: Supplementary file 5 — Additional file 5: Figure S1. miRNA-513a-3p exerts carcinogenic impact on PCa cells in vitro. (A) Relative expression of miR-513a-3p in PCa cell lines. (B) The relative abundance of miR-513a-3p in PC-3 and DU145 cells transfected with miR-513a-3p mimics or inhibitor. (C) The migration capability of PC-3 and DU145 cells transfected with miR-513a-3p inhibitor or inhibitor NC was measured by wound healing assays. The magnification is 100×. (D-G) The migration and invasion capabilities of PC-3 and DU145 cells transfected with miR-513a-3p inhibitor or inhibitor NC were assessed by Transwell migration and invasion assays. The magnification is 100×. (H, J) The proliferation ability of PC-3 and DU145 cells transfected with miR-513a-3p inhibitor or inhibitor NC was evaluated by CCK-8 assay. All data are expressed as means ± standard deviation. *P < 0.05, **P < 0.01 and ***P < 0.001. [file 12967_2023_4132_MOESM5_ESM.pdf]

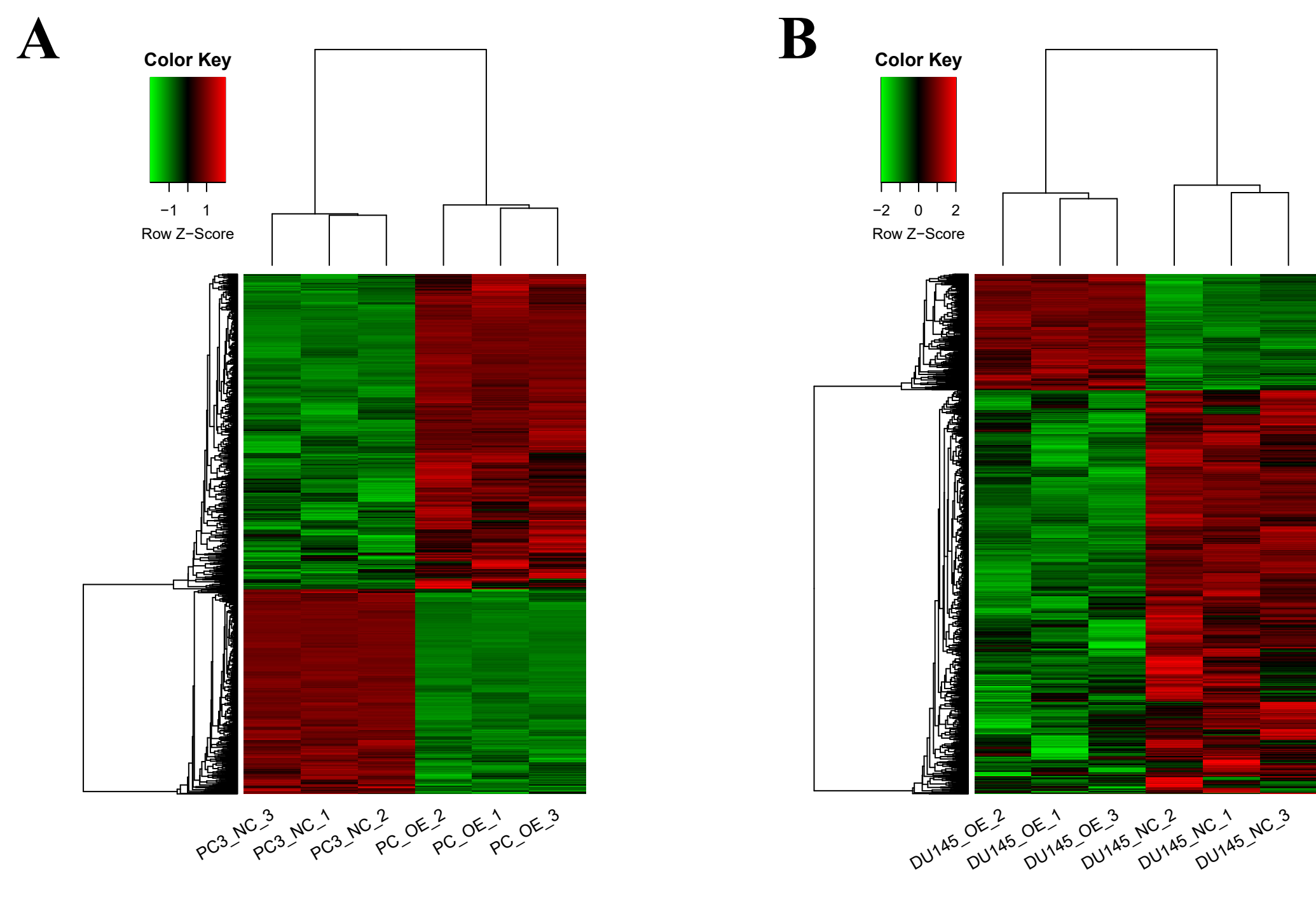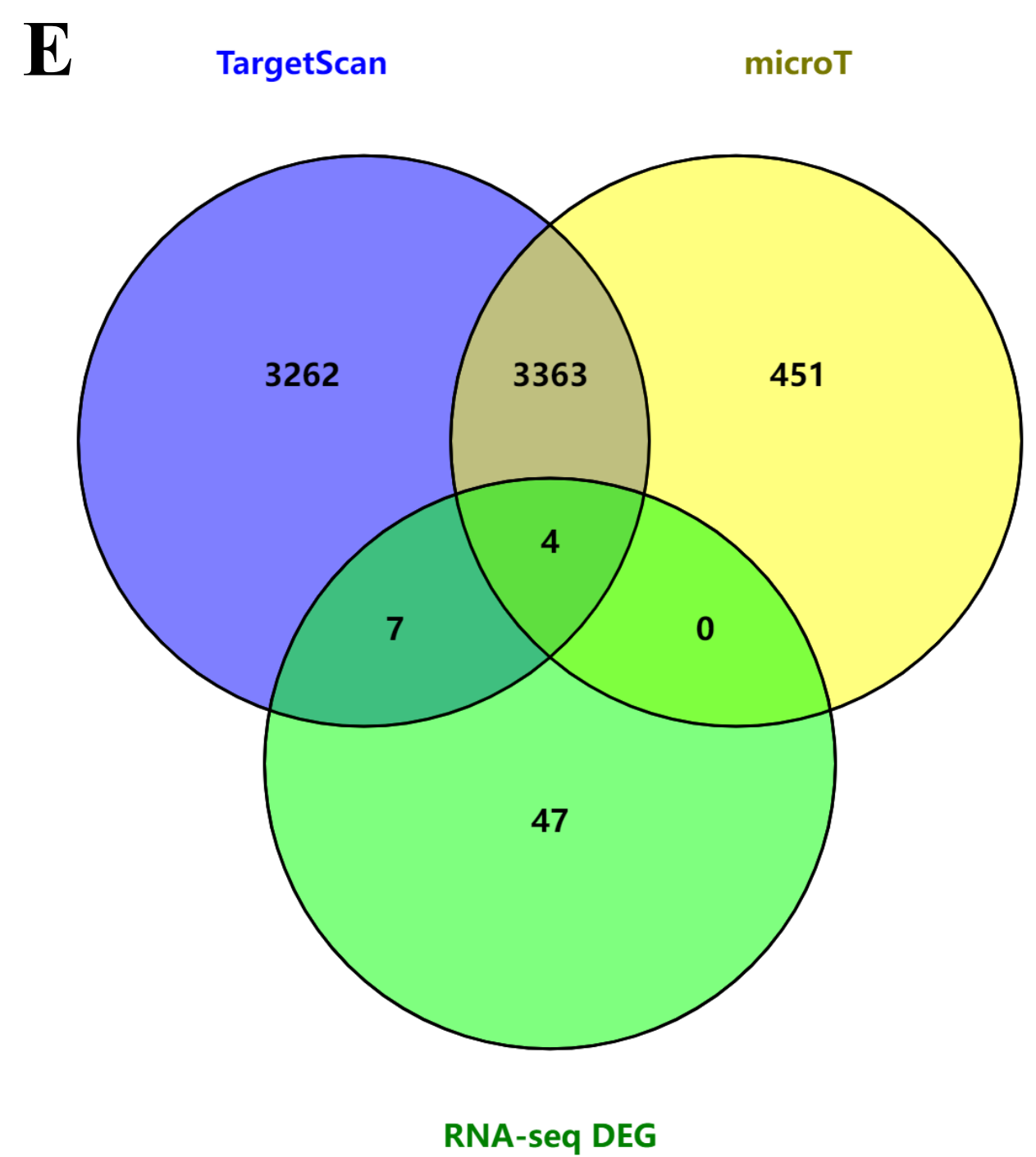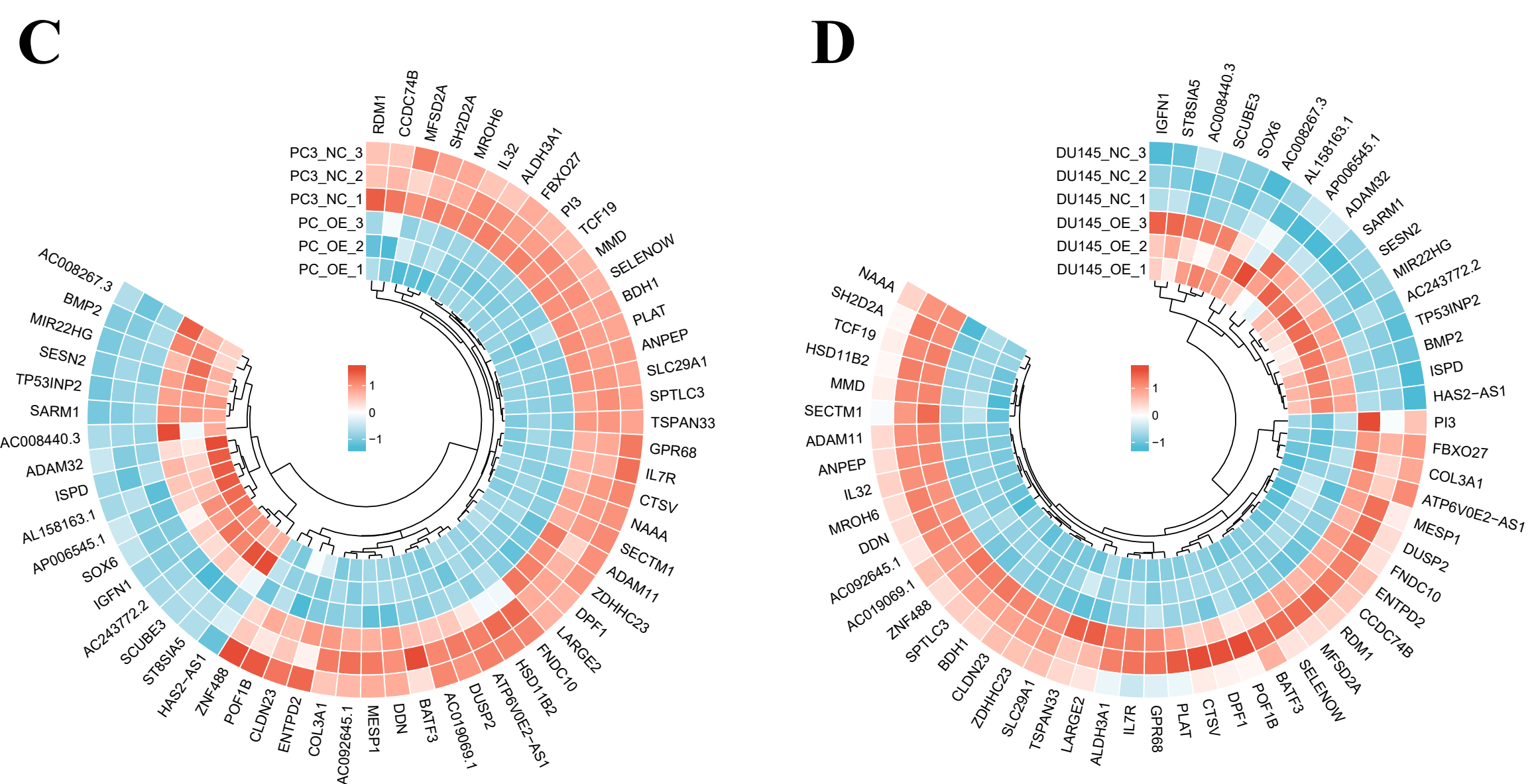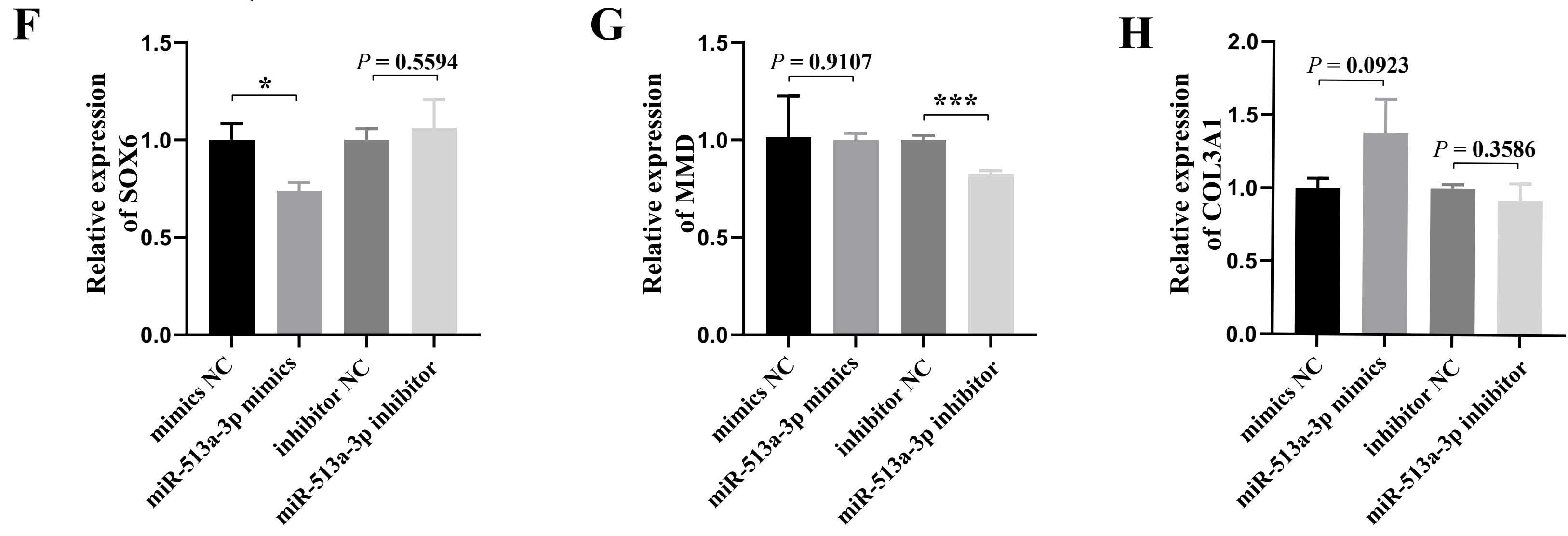

Supplement: Supplementary file 6 — Additional file 6: Figure S2. The screening of downstream target genes of miRNA-513a-3p. (A, B) Heat map of DEGs between circEPHA3-overexpressed and vector PCa cells. (C, D) Circos of 58 regulated DEGs in both PC-3 and DU145 cells. (E) Four candidate downstream target genes of circEPHA3/miR-513a-3p were predicted by TargetScan, microT and RNA-seq DEGs. (F-H) Relative expression of SOX6, MMD and COL3A1 in PCa cells after transfection of miR-513a-3p mimics, inhibitor and corresponding negative control. All data are expressed as means ± standard deviation. *P < 0.05, **P < 0.01 and ***P < 0.001. [file 12967_2023_4132_MOESM6_ESM.pdf]

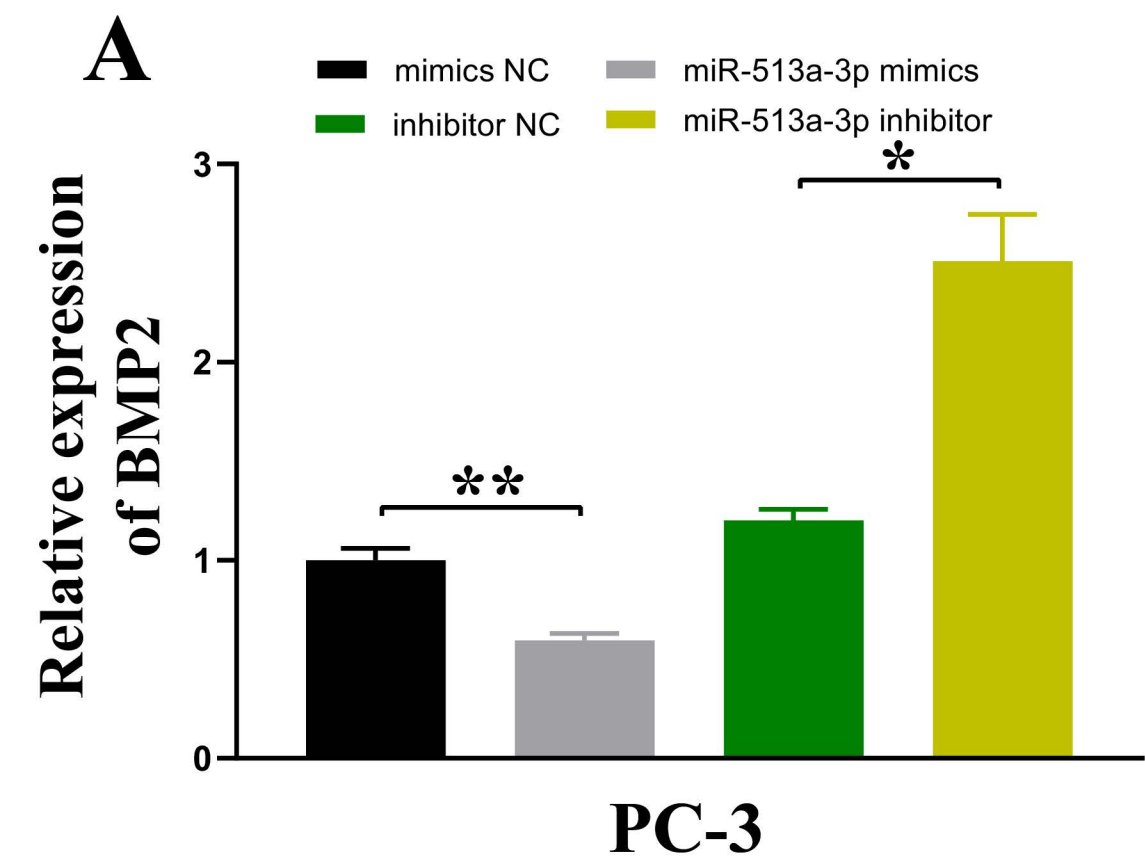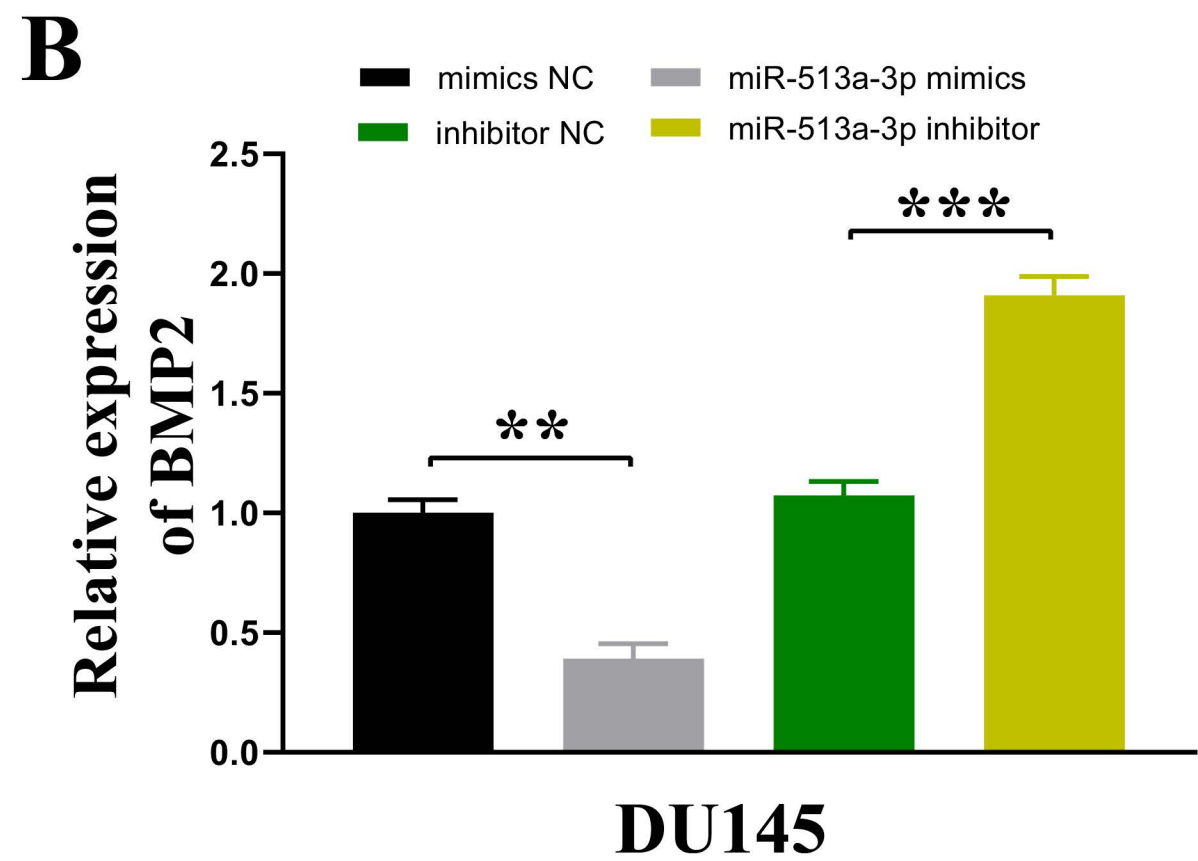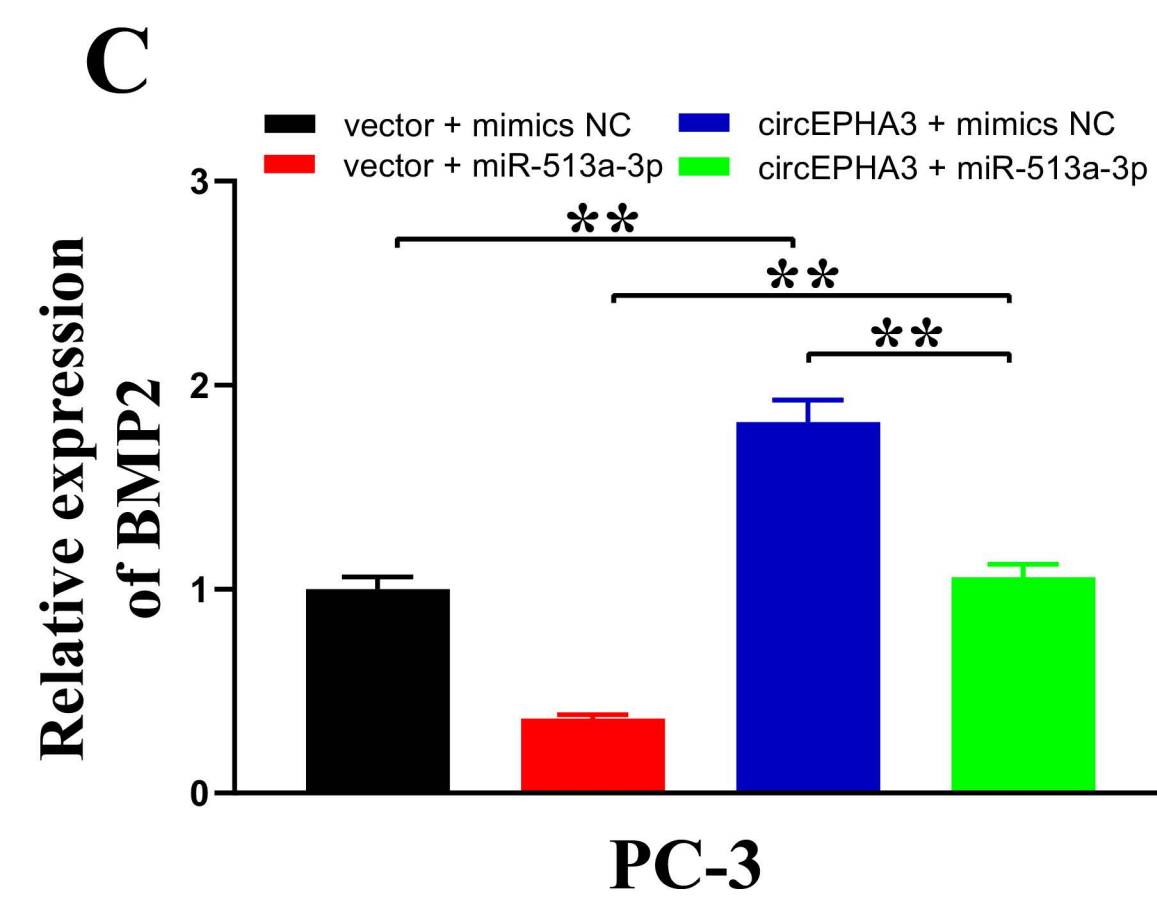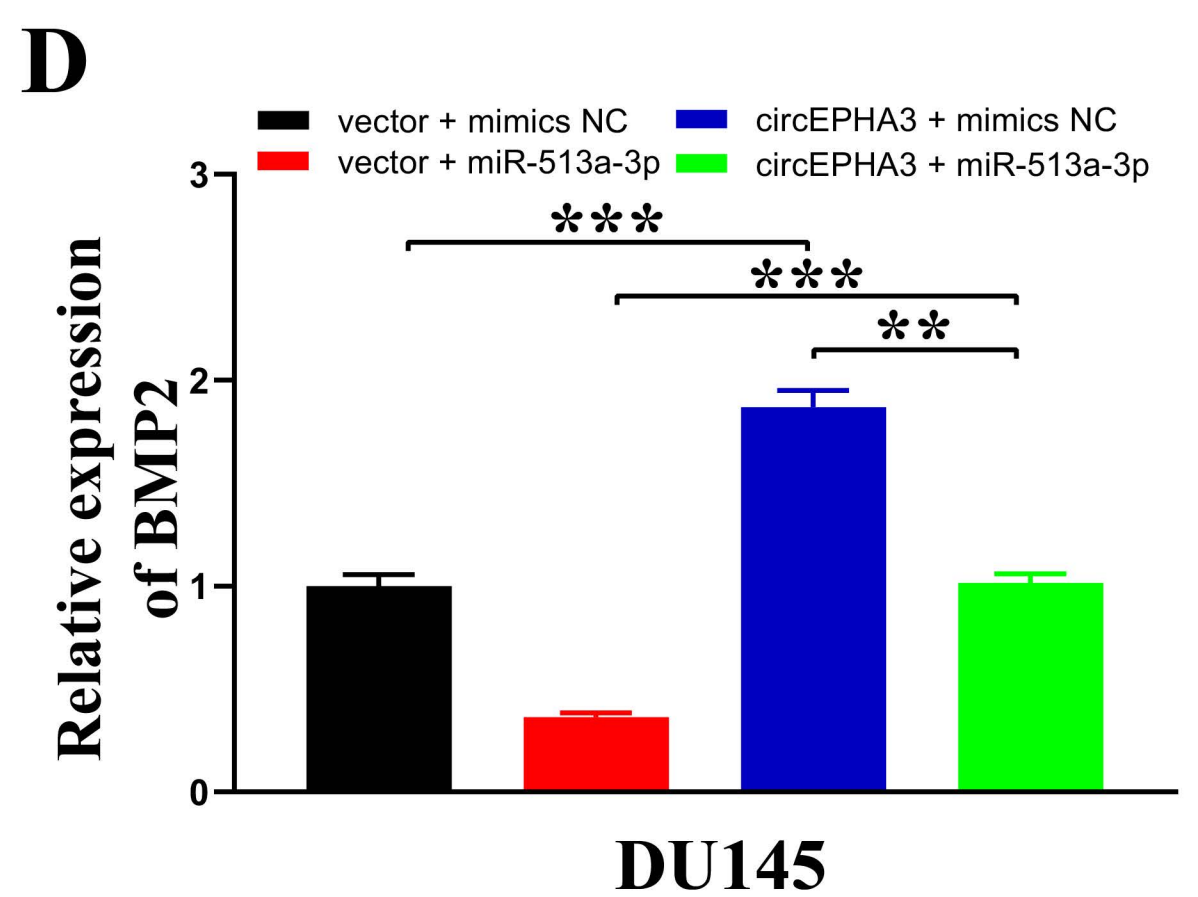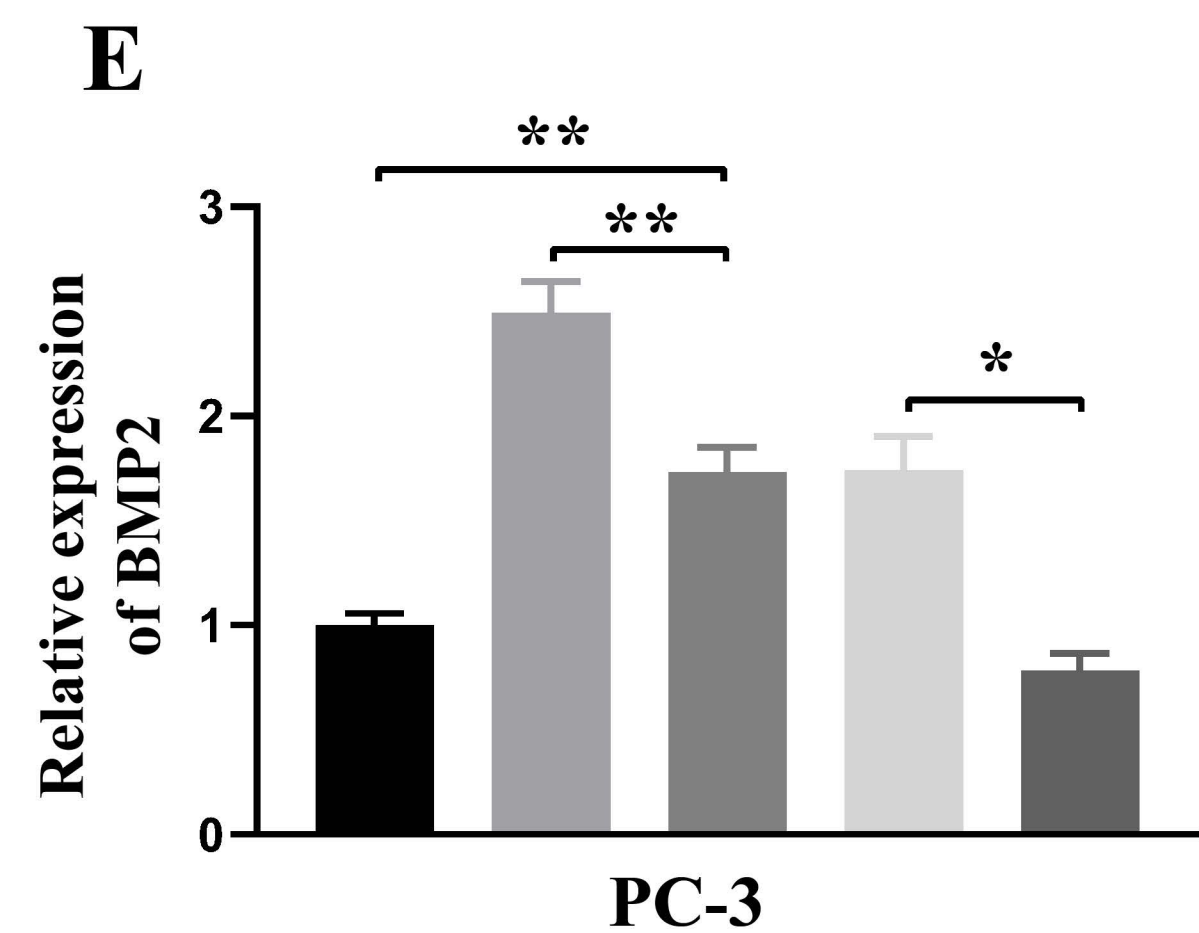

| Condition   | 1 | 2 | 3 | 4 | 5 |
|-------------|---|---|---|---|---|
| circEPHA3   | - | + | + | + | + |
| miR-513a-3p | - | - | - | + | + |
| si-BMP2     | + | - | + | - | + |

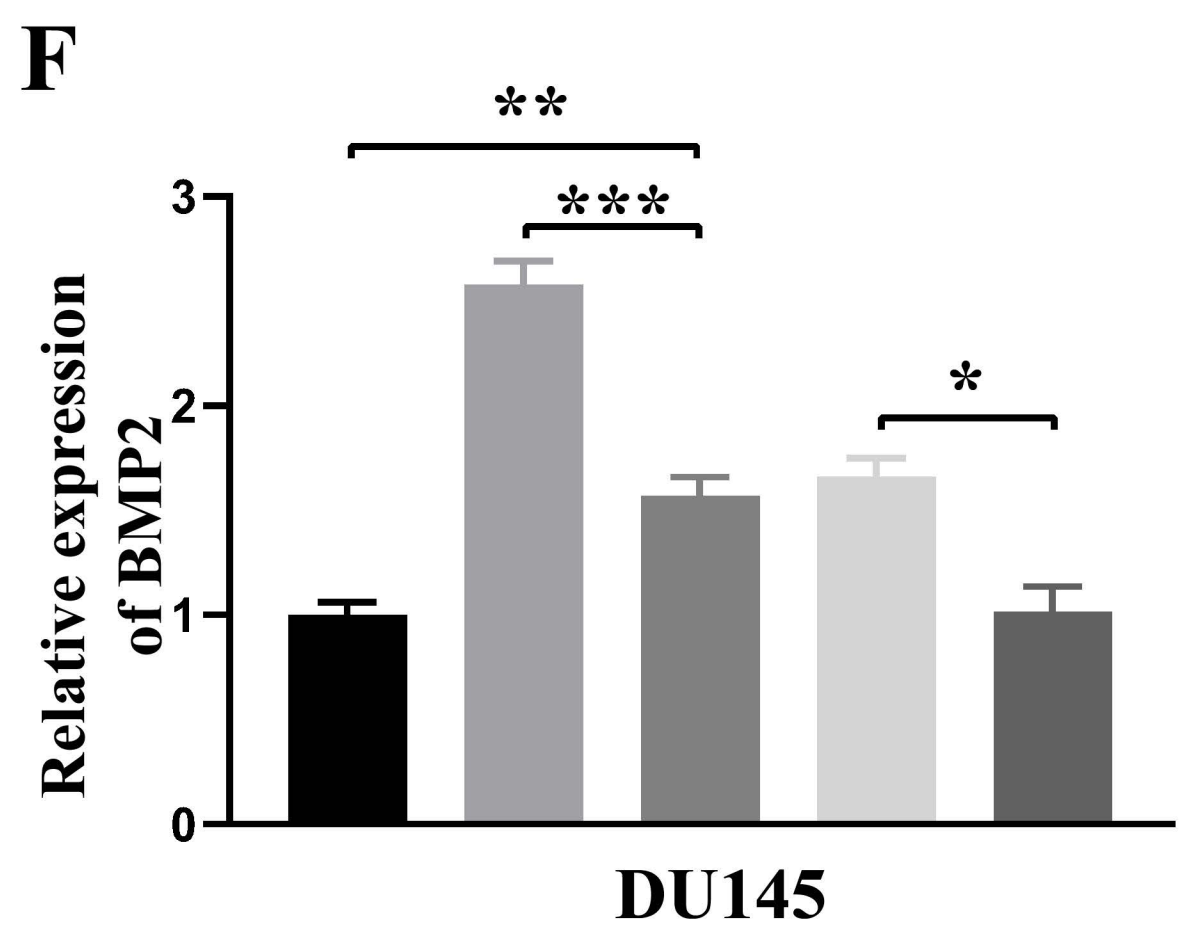

| Condition   | 1 | 2 | 3 | 4 | 5 |
|-------------|---|---|---|---|---|
| circEPHA3   | - | + | + | + | + |
| miR-513a-3p | - | - | - | + | + |
| si-BMP2     | + | - | + | - | + |

Supplement: Supplementary file 7 — Additional file 7: Figure S3. (A) The relative expression of BMP2 in PC-3 cells transfected with miR-513a-3p mimics and inhibitor. (B) The relative expression of BMP2 in DU145 cells transfected with miR-513a-3p mimics and inhibitor. (C) The relative expression of BMP2 in PC-3 cells transfected with miR-513a-3p mimics and cricEPHA3. (D) The relative expression of BMP2 in DU145 cells transfected with miR-513a-3p mimics and cricEPHA3. (E) The relative expression of BMP2 in PC-3 cells co-transfected with circEPHA3, miR-513a-3p and/or BMP2 siRNA. (F) The relative expression of BMP2 in DU145 cells co-transfected with circEPHA3, miR-513a-3p and/or BMP2 siRNA. All data are expressed as means ± standard deviation. *P < 0.05, **P < 0.01 and ***P < 0.001. [file 12967_2023_4132_MOESM7_ESM.pdf]

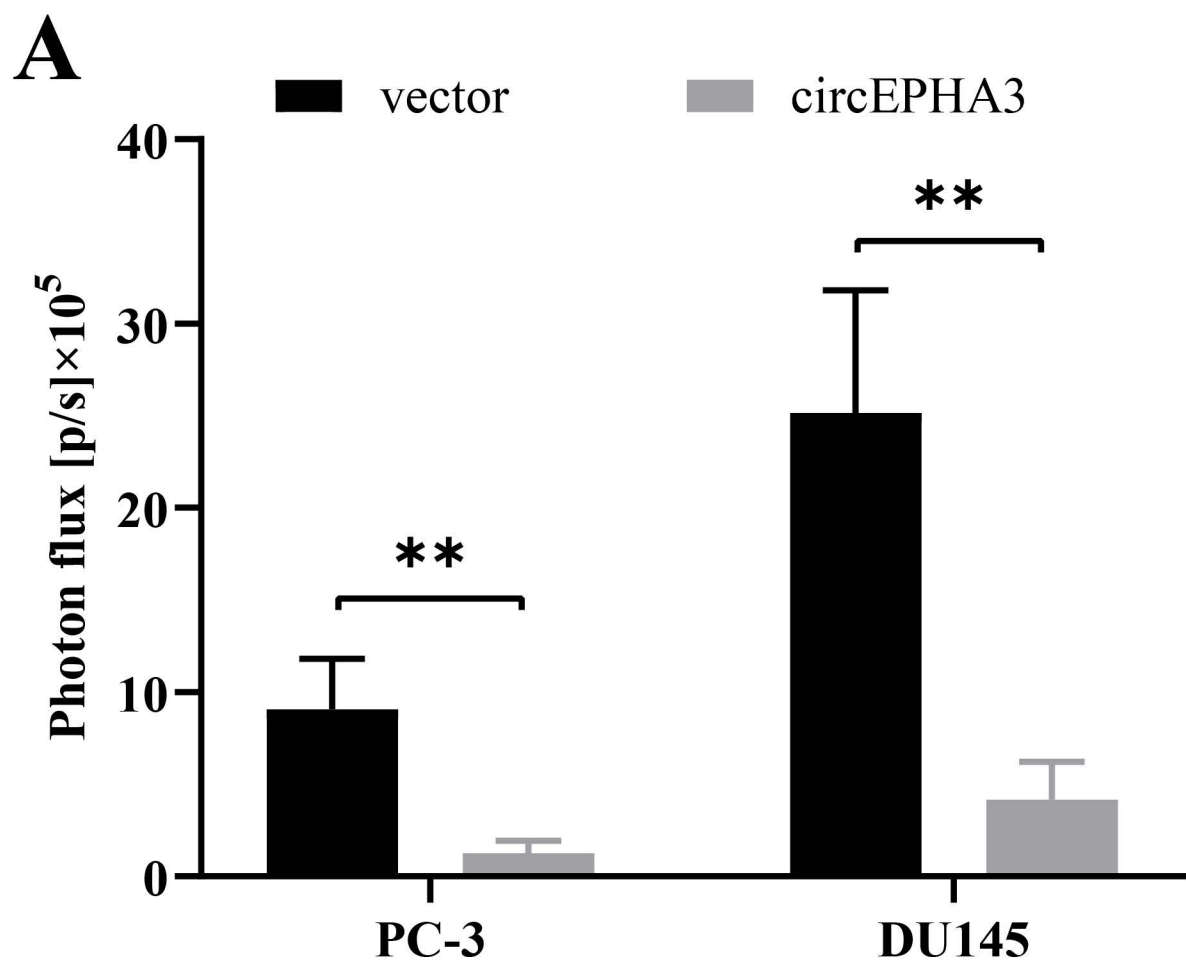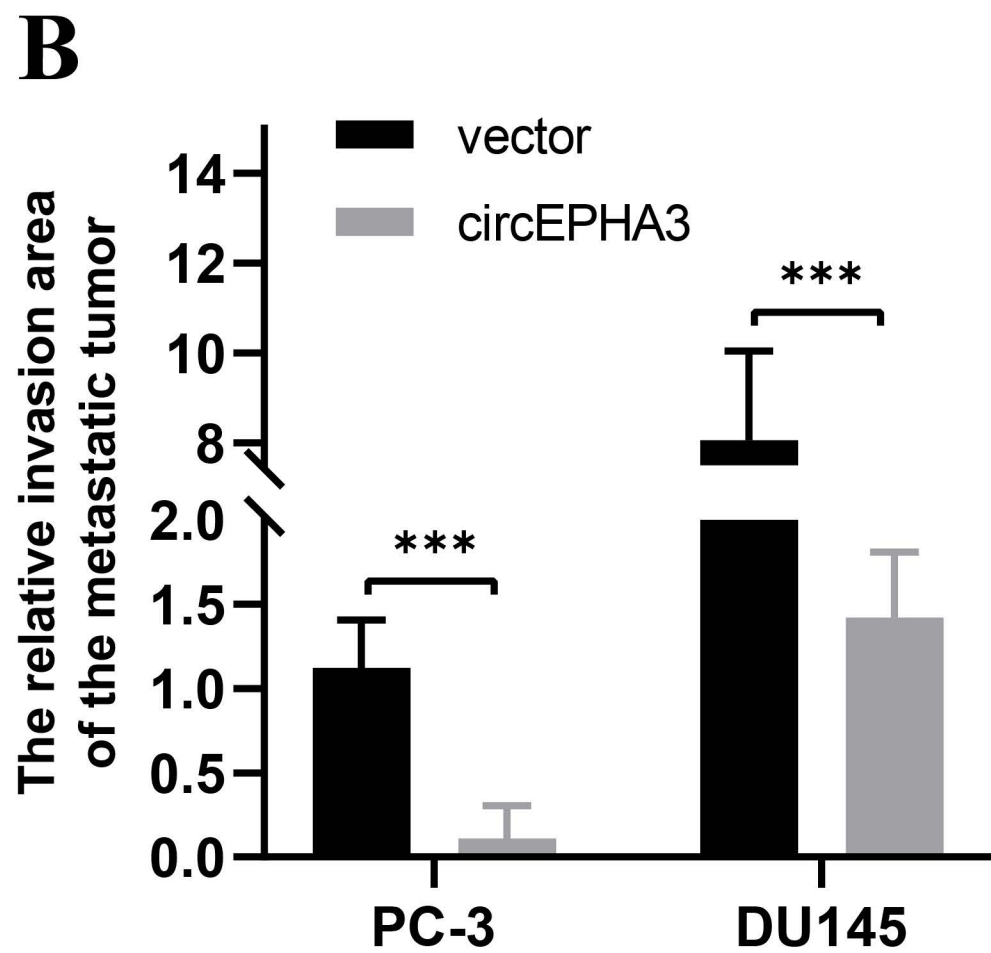

Supplement: Supplementary file 8 — Additional file 8: Figure S4. (A) The photon flux of each group of nude mice. (B) The relative invasion area of the metastatic tumor. All data are expressed as means ± standard deviation. **P < 0.01 and ***P < 0.001. [file 12967_2023_4132_MOESM8_ESM.pdf]
